# Innervation of the lophophore suggests that the phoronid *Phoronis ovalis* is a link between phoronids and bryozoans

**DOI:** 10.1038/s41598-017-14590-8

**Published:** 2017-10-31

**Authors:** Elena N. Temereva

**Affiliations:** 1 Department Invertebrate Zoology, Biological Faculty, Moscow State University, 119991 Moscow, Russia; 2 Biological Faculty, Moscow State University, Leninskie Gory, 1-12, 119991 Moscow, Russia

## Abstract

The validity of the Lophophorata as a monophyletic group remains controversial. New data on the innervation of the lophophore, which is a unique feature of the lophophorates, may help clarify the status of the Lophophorata and provide new information on the early evolution of the group. In this paper, the organization of the nervous system of the lophophore is described in adults of the minute phoronid *Phoronis ovalis*. The lophophore nervous system includes a dorsal ganglion, a tentacular nerve ring, an inner ganglion, an inner nerve ring, and six nerves in each tentacle. The inner ganglion and inner nerve ring, which is associated with sensory cells, are described for the first time in adult phoronids. The general plan of the nervous system of the lophophore and tentacles is similar in *P. ovalis* and bryozoans. These new results suggest the presence of two nerve centers and two nerve rings in the last common ancestor of phoronids and bryozoans. During evolution, bryozoans may have lost the outer nerve center and outer nerve ring, whereas phoronids may have lost the inner nerve center and inner nerve ring. These morphological results evidence the lophophorates are monophyletic.

## Introduction

Tentacular apparatuses are known in many different diploblastic and bilaterian animals. The lophophore is specific tentacular apparatus, in which tentacles surround the mouth but not the anus. Based on their possession of a lophophore, three phyla of invertebrates have been traditionally regarded as lophophorates: phoronids, brachiopods, and bryozoans (=ectoprocts)^1–4^. Although the monophyly of lophophorates has been supported by anatomical and embryological data^1–6^ and by some molecular phylogenetic data^7,8^, most molecular data are inconsistent with the unity of the lophophorates^9,10^. Accordingly to many molecular results, the lophophorates have been divided into two unrelated clades: Brachiozoa (Phoronida + Brachiopoda) and Bryozoa^10–12^.

At the same time, bryozoans have a lot in common with phoronids. Both groups have a similar bauplan: the dorsal side of adult animals is very short but the ventral side is very long; the digestive tract is U-shaped: mouth and anus are placed closely each other^13^. Phoronids are sessile benthic animals living in organic tubes, which are embedded in soft or hard substrata. The ventral portion of the phoronid body is embedded in the tube, while the lophophore and associated tentacles extend into the water. When in danger, the adult phoronid strongly contacts its longitudinal muscles, causing the body length to greatly decrease and the lophophore to be pulled into the tube. Some phoronids are known to reproduce asexually, which leads to the formation of a “colony”^4^. Most adult bryozoans are colonial animals. Each bryozoan individual (zooid) in a colony consists of two parts: a soft polypide with the lophophore and tentacles, and a cystid, which is the firm shelter for the internal organs. When in danger, the zooid pulls the polypide with the lophophore into the cystid.

Although the general morphology of the lophophore is similar among different lophophorates, it differs in detail between and within phyla^3,14,15^. The lophophore has two morphological schemes. In one scheme, which is characteristic of all brachiopods, new tentacles arise at the tip of each lophophore arm. In the second scheme, which is characteristic of both phoronids and bryozoans, new tentacles arise at two points located behind the mouth. The scheme of the lophophore organization is another feature that is similar between phoronids and bryozoans.

Phoronids have six main types of lophophore organization^3,15^. The oval lophophore is the simplest type, and *Phoronis ovalis* has an oval lophophore that bears about 20 tentacles. According to molecular data, *P. ovalis* is regarded as the sister taxon to the remaining Phoronida^16^ and the oval lophophore is supposed to be the ancestral type for all phoronids^3^. Among bryozoans, there are four main types of lophophore. The complex horseshoe-shaped lophophore bears more than 40 tentacles and is known in some recent phylactolaemates. The simplest lophophore is round and occurs in many bryozoans. The evolution of the lophophore within bryozoans has been discussed^6,17^.

Innervation of the lophophore and tentacles seems to differ among different lophophorates. At the same time, some lophophoral main nerves can be regarded as homologous in all lophophorates^5,6,18^. According to previous results^5,6^, all lophophorates have three main nerve centers: the dorsal (=cerebral) ganglion, the inner lophophoral nerve ring, and the outer lophophoral nerve ring. In brachiopods, there is tendency for the inner lophophoral nerve ring to be weakly developed. This tendency was recently described in two brachiopod species, which belong to two different classes, but have the same type of the lophophore^19^. Juvenile phoronids have an inner lophophoral nerve ring^20,21^, but adult phoronids do not^22–24^. Most species of bryozoans have only the inner lophophoral nerve ring; the outer lophophoral nerve ring has been described only in *Amathia gracilis* ^6^.

The investigation of lophophore innervation in additional specimens from each phylum of the lophophorates may help clarify the homology of the lophophore and may provide insight into the question of lophophorates monophyly. For these reasons, the study of lophophore innervation in adult phoronids could be very useful.

This report studies the nervous system of the lophophore and tentacles in adults of the minute phoronid *Phoronis ovalis*. As noted earlier, these results could help answer the question on homology of lophophores within the lophophorates and decide whether the lophophorates are monophyletic.

## Results

### General morphology of *Phoronis ovalis*

**Figure 1:**
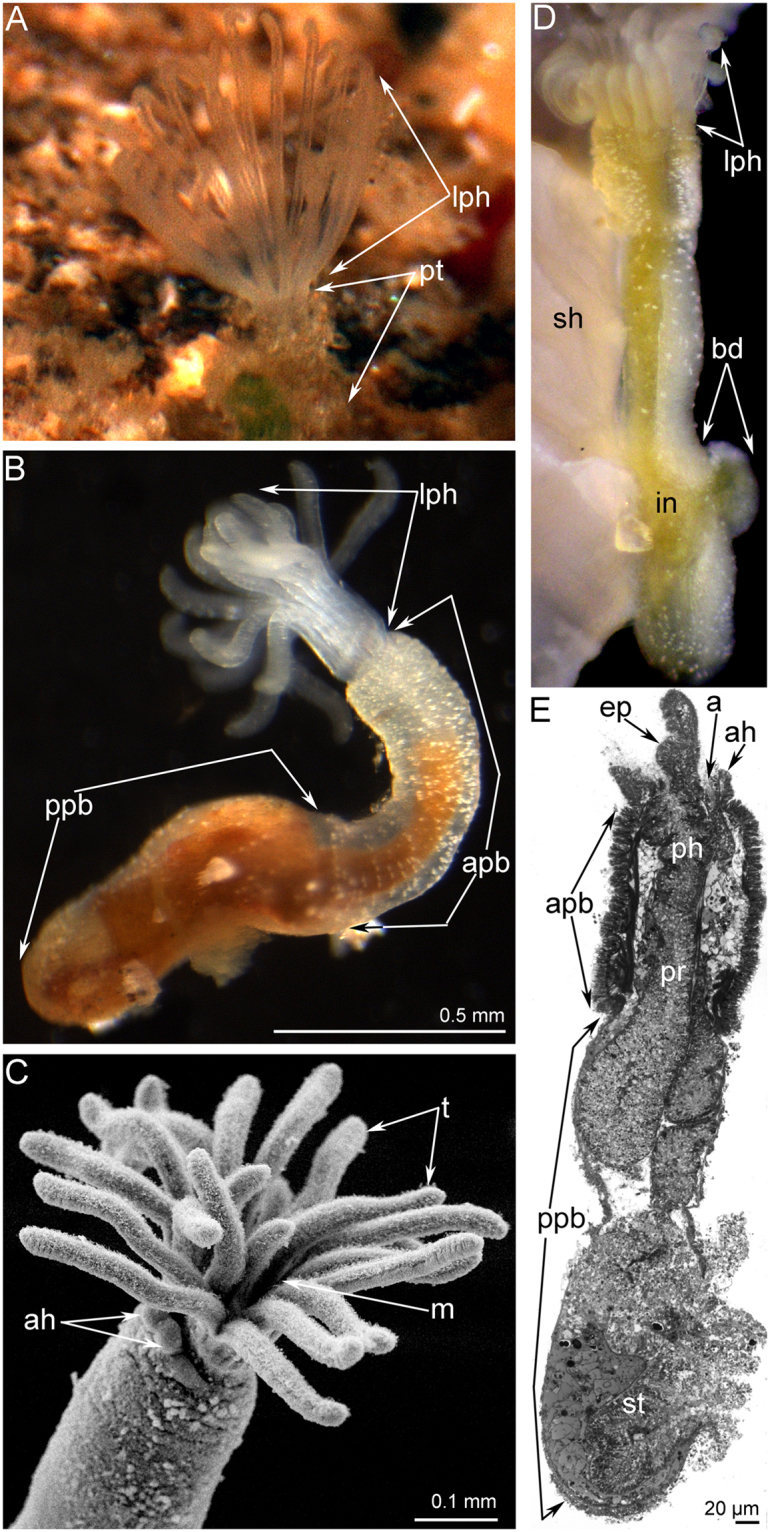
General morphology of *Phoronis ovalis*. (**A**) the lophophore of live animal extended from the tube, which is embedded into the shell, (**B**) whole live animal viewed from the right, (**C**) the lophophore viewed from the top, SEM, (**D**) whole animal, which is embedded into the shell (sh) and forms a bud (bd), (**E**) sagittal semithin section of whole animal: the difference between anterior and posterior parts of the body is evident. Abbreviations: a, anus; ah, anal hill; apb, anterior part of the body; bd, bud; ep, epistome; in, intestine; lph, lophophore; m, mouth; ph, pharynx; ppb, posterior part of the body; pr, prestomach; st, stomach; pt, portion of tube; sh, shell; t, tentacle.

**Figure 2:**
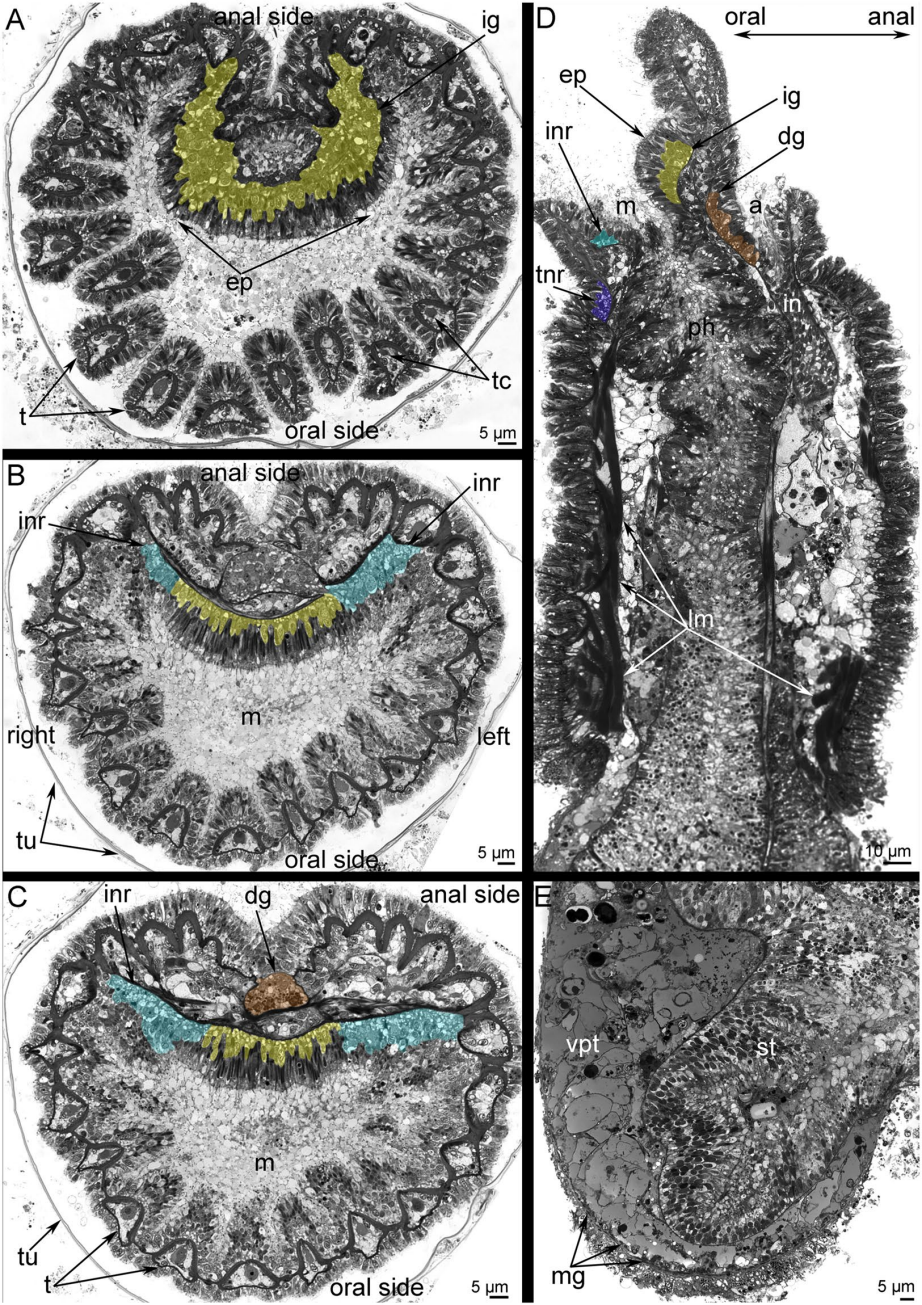
Location of the main nerve elements of the lophophore of *Phoronis ovalis* (semithin sections). (**A**) transversal section of the epistome middle, (**B**) transversal section of the epistome base, (**C**) transversal section of the dorsal ganglion, (**D**) sagittal section of the anterior portion of body, (**E**) sagittal section of the posterior portion of body. Abbreviations: a, anus; ep, epistome; dg, dorsal ganglion; ig, inner ganglion; in, intestine; inr, inner nerve ring; lm, longitudinal muscles; m, mouth; ph, pharynx; st, stomach; t, tentacle; tc, tentacle capillary; tnr, tentacular nerve ring; tu, tube; vpt, vasoperitoneal tissue.

*Phoronis ovalis* is a very small phoronid that burrows into the shells of bivalves. The lophophore and a portion of the body are exposed in the water (Fig. 1A). The oval-shaped lophophore bears 24 tentacles (Fig. 2A–C). The tentacles surround the mouth, which is partly covered at the dorsal side by the epistome. The epistome is a linguiform epidermal fold with a compact, thick tip (Fig. 2A) and a wide base (Fig. 2B–D). The integument of live animals is transparent and has many white spots (Fig. 1B,D). The coloured digestive tract is visible through the integument (Fig. 1B,D). The lophophore is separated from the body by a circular groove (Fig. 1B,C). The bilobed anal hill is located near this groove, on the anal side of the proximal portion of the anterior body (Fig. 1C). The anus opening is at the top of this hill (Fig. 2D). The body is worm-like and divided into several parts. The anterior part of the body is one-half of the body length; it is characterized by a thick epithelium and prominent bundles of longitudinal muscles (Figs 1E and 2D). In some specimens, the proximal portion of the anterior body part is swollen (Fig. 1D). A border is evident between the anterior and posterior parts of the body: the longitudinal muscles abruptly end at this border. The posterior part of the body is characterized by a thin integument and by a lack of prominent bundles of longitudinal muscles. The coelom of the posterior part of the body contains vasoperitonal tissue (Fig. 2E). The posterior part is not divided externally. However, the distal (terminal) portion of the posterior part has a prominent muscle grid. In some specimens, the posterior part of the body forms a bulb (Fig. 1D). This is a bud resulting from asexual reproduction. The digestive tract forms a branch that penetrates this bud.

### Serotonin-like immunoreactive (5ht-lir) nerve elements of the lophophore

**Figure 3:**
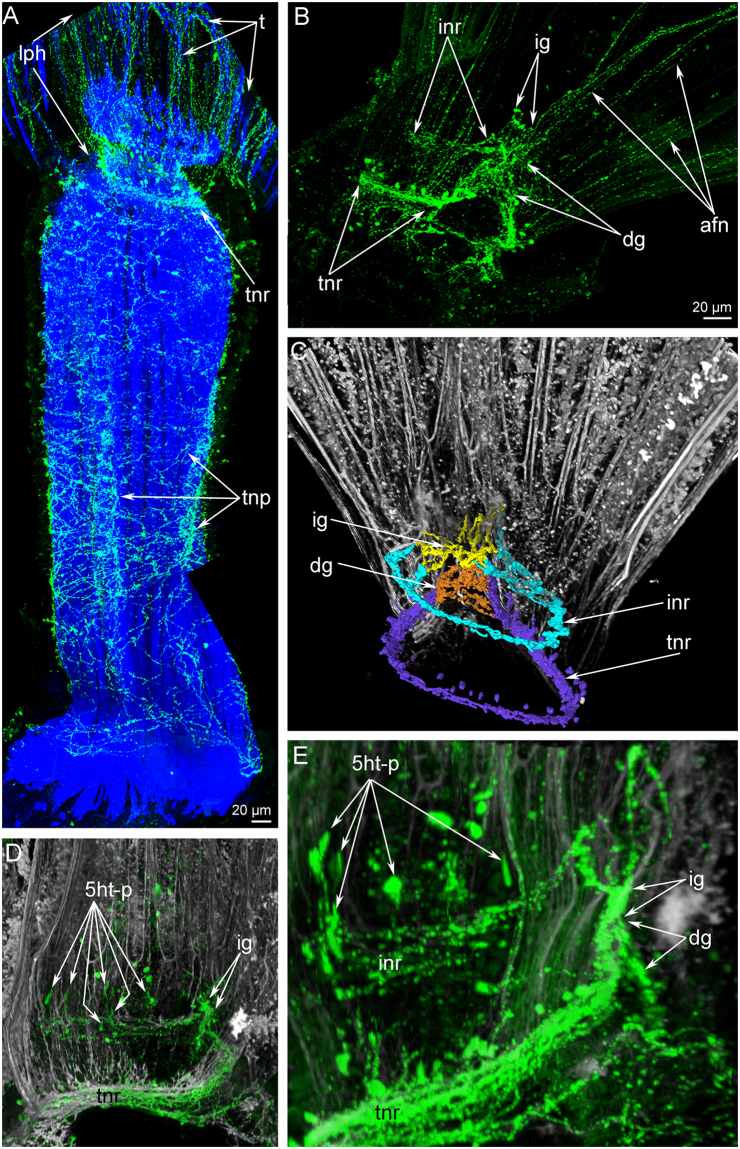
Serotonin-like immunoreactive nerve elements of the lophophore of *Phoronis ovalis*. Z-projections (**A**,**B**) and 3D-reconstructions (C-E) after mono- and double staining for 5-HT (serotonin) (green), acetylated α-tubulin (grey), and phalloidin (blue). (**A**) general view of serotonin-like immunoreactive nerve net developed in the anterior portion of the body and the lophophore, (**B**) main nerve elements viewed from the anal side, (**C**) reconstruction of inner nerve centre with inner nerve ring and dorsal ganglion with tentacular nerve ring, (**D**) inner nerve ring viewed from the left. The left side of the lophophore is deleted, (**E**) connection between inner ganglion and dorsal ganglion viewed from the left. Abbreviations: 5ht-p, serotonin-like immunoreactive perikarya connected with the inner nerve ring; afn, abfrontal neurite bundle; dg, dorsal ganglion; ig, inner ganglion; inr, inner nerve ring; lph, lophophore; t, tentacle; tnr, tentacular nerve ring.

**Figure 4:**
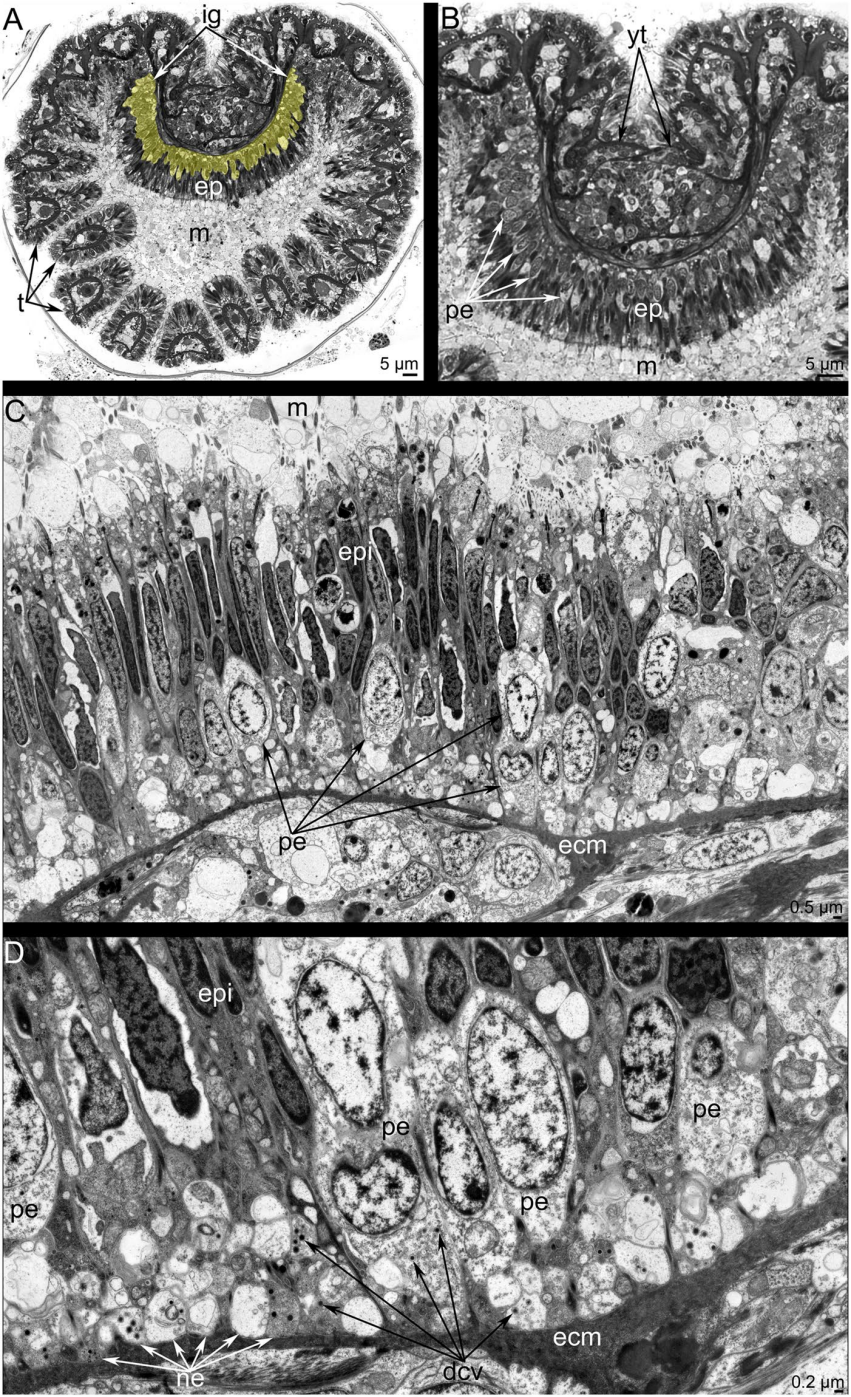
Organization of the inner ganglion in *Phoronis ovalis*. Semithin (**A**,**B**) and ultrathin (**C**,**D**) transversal sections. (**A**) section of the epistome tip with the inner ganglion, (**B**) the location of the inner ganglion in the epithelium of the epistome, (**C**) perikarya of inner ganglion scattered within epithelium of the epistome, (**D**) ultrastructure of neurites and perikarya: cytoplasm is filled with dense-core vesicles, and synaptic vesicles with electron light content. Abbreviations: dcv, dense-core vesicles; ecm, extracellular matrix; epi, epithelium; ig, inner ganglion; m, mouth; ne, neurite; pe, perikaryon; t, tentacle; yt, youngest tentacles.

**Figure 5:**
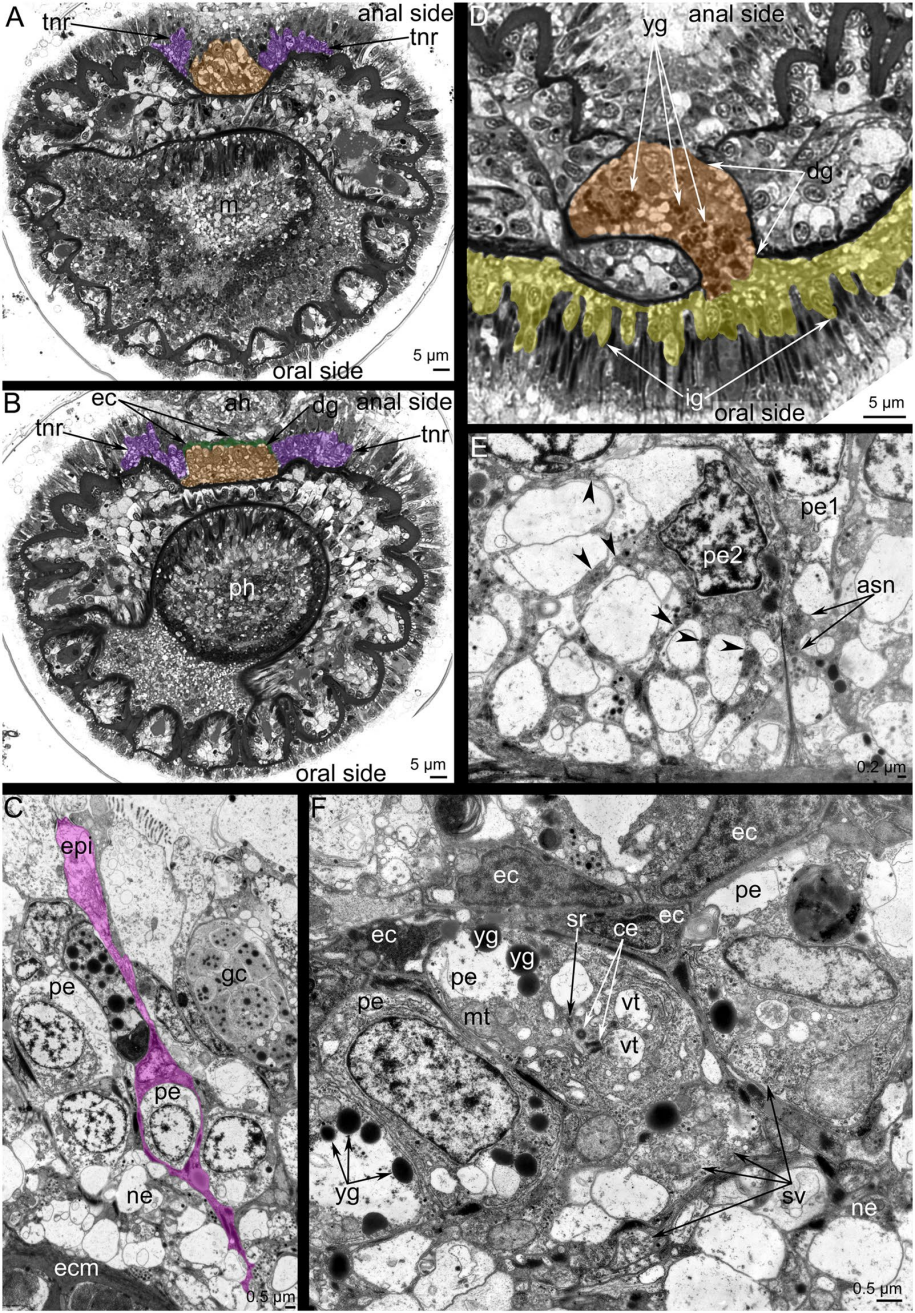
Organization of the dorsal ganglion and tentacular nerve ring in *Phoronis ovalis*. Semithin (**A**,**B**,**D**) and ultrathin (**C**,**E**,**F**) transversal sections. (**A**) Section of the tip of the dorsal ganglion (brown), (**B**) section of the dorsal ganglion (brown) base: the layer of envelope cells (ec) is marked by green, (**C**) at the tip of dorsal ganglion, perikarya are surrounded by thin projections of envelope cells (pink), (**D**) the connection between the dorsal ganglion (brown) and inner ganglion (yellow); (**E**) a portion of the tentacular nerve ring: perikarya of second type form thin projections (arrowheads), which form bundles (asn), (**F**) organization of perikarya of middle zone of the dorsal ganglion: perikarya contain yolk granules (yg), centrioles (ce), and synaptic vesicles (sv). Abbreviations: ah, anal hill; ce, centriole; dg, dorsal ganglion; ec, envelope cell, ecm, extracellular matrix; epi, epithelium; gc, gland cells; ig, inner ganglion; m, mouth; mt, mitochondria; ne, neurite; pe, perikaryon; pe1, perikaryon of first type; pe2, perikaryon of second type; ph, pharynx; sv, synaptic vesicle; tnr, tentacular nerve ring; vt, multivesicular body; yg, yolk granule.

In *P. ovalis*, the 5ht-lir neurites and perikarya form a net around the anterior part of the body and around the lophophore base (Fig. 3A). The main nerve elements are located in the lophophore (Fig. 3B–D). The lophophore contains four main 5ht-lir nerve elements. The first element is the inner nerve centre (inner ganglion), which is located on the oral side of the epistome. At the epistome tip, the inner ganglion is represented by a large aggregation of perikarya and neurites, which form a horseshoe-like structure (Fig. 2A). At the epistome base, the shape of the inner ganglion changes: it looks like a long row of nerve cells, which are embedded in the epithelium of the epistome (Figs 2B,C and 4A). The second 5ht-lir main nerve element is the inner nerve ring (inner lophophoral nerve), which starts from the inner nerve centre and extends around the mouth, at the base of the frontal side of the tentacles (Figs 2D and 3C,D). The third 5ht-lir main nerve element is the dorsal ganglion (=dorsal nerve plexus). It is placed on the dorsal side of the body, in the lophophoral concavity, between the youngest tentacles and the anus (Fig. 2C,D).The aggregation of perikarya, which form the dorsal ganglion, is pyramidal with a small tip (Fig. 5A) and a wide base (Fig. 5B). The dorsal ganglion gives rise to the tentacle nerve ring (outer lophophoral nerve), which is the fourth 5ht-lir main nerve element. The tentacle nerve ring passes from the dorsal ganglion by two branches (Fig. 5A,B) and extends at the base of the lophophore along the abfrontal sides of tentacles. The inner ganglion and the dorsal ganglion connect (Fig. 5D), in some cases forming a compact X-like structure that gives rise to both inner and outer lophophoral nerves (Fig. 3E).

### Ultrastructure of the main nerve elements

The ultrastructure of the inner ganglion and the inner nerve ring is similar. Both nerve elements are formed by perikarya and neurites, which are scattered in the epithelium of the epistome (Fig. 4A). Perikarya are located between basal projections of epithelial cells and form a thick basal layer under the bodies of the epithelial cells (Fig. 4B,C). Most perikarya are large cells with electron-lucent cytoplasm and a roundish nucleus that contains a nucleolus (Fig. 4D). The cytoplasm of perikarya contains synaptic vesicles, most of which are dense-core vesicles (Fig. 4D). Neurites form a thick basal layer between the extracellular matrix and the soma of perikarya. There are two main types of neurites. One type is characterized by electron-lucent cytoplasm and the presence of mostly dense-core vesicles. The second type has electron-dense cytoplasm and contains electron-lucent synaptic vesicles and synaptic vesicles with electron-dense middles (Fig. 4D).

The dorsal ganglion is an aggregation of large perikarya, whose ultrastructure differs at different levels of the dorsal ganglion. Thus, large perikarya with electron-lucent cytoplasm and large, round nuclei (one per perikaryon) and nucleoli (one per nucleus) form the tip of the dorsal ganglion. These perikarya are surrounded by long, thin projections of epithelial cells (Fig. 5C). In the middle and basal portions of the ganglion, small cells form a layer between the soma of epithelial cells and the soma of perikarya (Fig. 5B,F). These small cells (envelope cells) have electron-dense cytoplasm and small nuclei (one per cell) with electron-dense chromatin (Fig. 5F). The middle portion of the ganglion is formed by perikarya with dense cytoplasm that contains many electron-dense granules (yolk granules) that can be recognized in semi-thin sections (Fig. 5D) and in thin sections (Fig. 5F). The cytoplasm is filled with rough endoplasmic reticulum, multi-vesicular bodies, mitochondria, and synaptic vesicles of different sizes (Fig. 5F). In some perikarya, a basal body, an additional centriole, and a short striated root are evident (Fig. 5F). The basal portion of the dorsal ganglion has a similar organization as the middle portion but does not contain yolk granules. In the dorsal ganglion, most neurites have large diameter and electron-lucent cytoplasm.

The tentacle nerve ring is formed by two types of perikarya. The ultrastructure of the first type is similar to that of perikarya of the dorsal ganglion. The perikarya of the second type are small and have electron-dense cytoplasm and small, irregularly shaped nuclei (Fig. 5E). The perikarya of the second type form long, thin projections that form an aggregation that passes between neurites of large diameter (Fig. 5E).

### Innervation of tentacles

**Figure 6:**
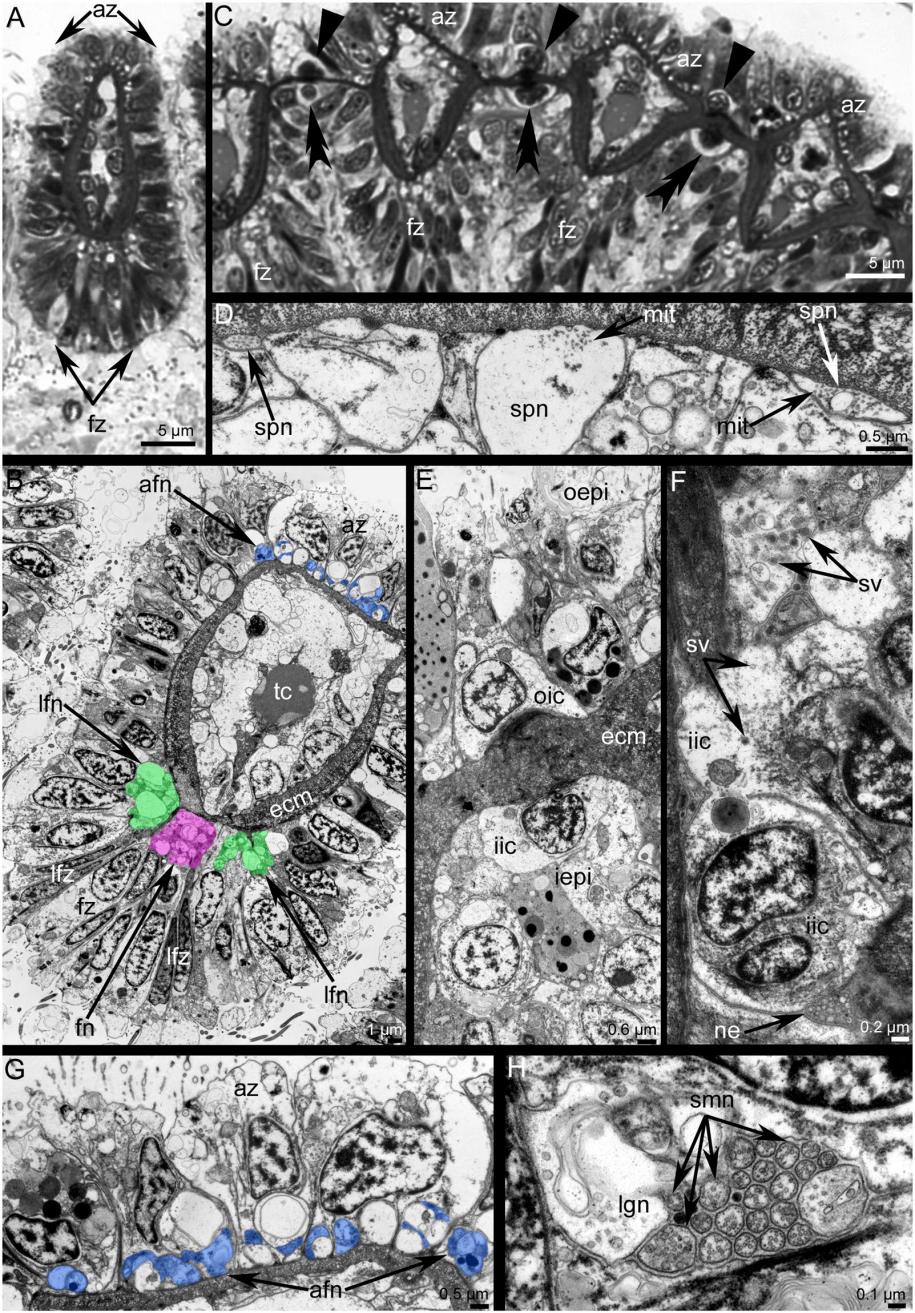
Innervation of tentacles in *Phoronis ovalis*. Semithin (**A**,**C**) and ultrathin (**B**,**D**–**H**) transversal sections. (**A**) A shape of tentacles and location of main zones, (**B**) location of tentacular nerves in epithelium: abfrontal nerves are in blue, frontal nerve is in pink, laterofrontal nerves are in green, (**C**) row of tentacles with large cells between them, which form outer (straight arrowheads) and inner (double arrowheads) rows, (**D**) subperitoneal neurites, (**E**) outer and inner rows of inter-tentacular large cells, (**F**) ultrastructure of inner inter-tentacular cell, (**G**) abfrontal neurite bundles, (**H**) laterofrontal neurite bundle consists of 20 neurites of small diameter and one large neurite. Abbreviations: afn, abfrontal neurite bundle; az, abfrontal zone; ecm, extracellular matrix; fn, frontal neurite bundle; fz, frontal zone; iepi, inner epithelium of tentacles; iic, inner inter-tentacular cell; lfn, laterofrontal neurite bundle; lfz, laterofrontal zone; lgn, neurite of large diameter; mit, microtubules; ne, neurite; oepi, outer epithelium of tentacles; oic, outer inter-tentacular cell; smn, neurite of small diameter; spn, subperitoneal neurite; v, synaptic vesicle; tc, tentacular capillary.

**Figure 7:**
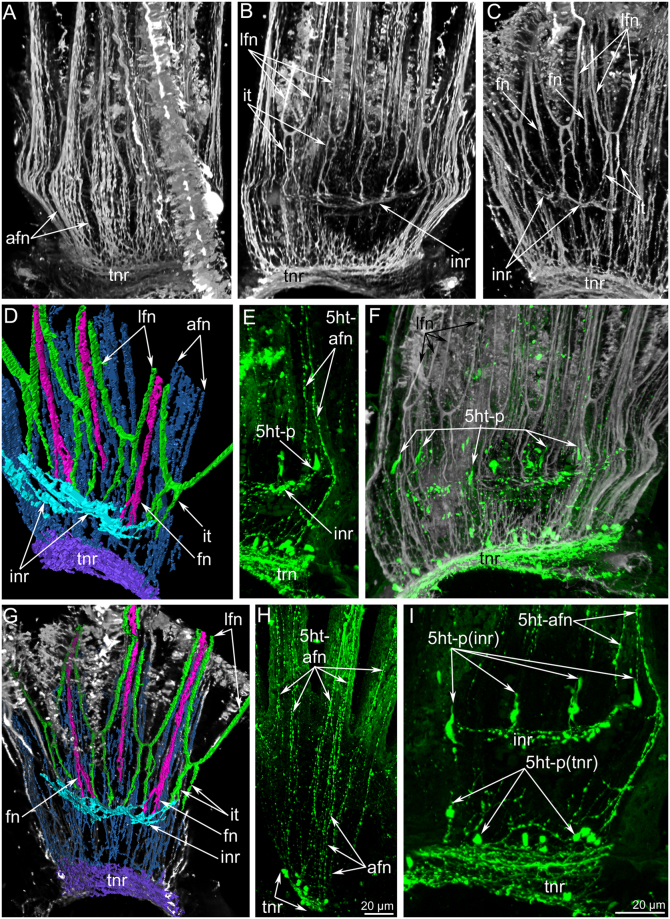
Innervation of tentacles in *Phoronis ovalis* accordingly to data on cytochemistry and laser confocal scanning microscopy. Three-dimensional reconstructions (**A**–**G**) and Z-projection (**H**,**I**) after mono- and double staining for 5-HT (serotonin) (green) and acetylated α-tubulin (grey). (**A**) abfrontal zones of tentacles: numerous thin neurites extend from the tentacular nerve ring along abfrontal side of each tentacle, (**B**) paired inter-tentacular nerves extend from the inner nerve ring, (**C**) frontal tentacular nerves extend from the inner nerve ring, (**D**) reconstruction of all nerves in several tentacles, (**E**) two lateroabfrontal neurites exhibit 5ht-like immunoreactivity, (**F**) 5ht-like immunoreactive perikarya connect the inner nerve ring and are located at the tentacles base between inter-tentacular nerves, (**G**) reconstruction of tentacular nerves and their connection with the inner nerve ring and tentacular nerve ring, (**H**) 5ht-like immunoreactive abfrontal neurites, which are numerous near the tentacular nerve ring and are presented as two bundles in each tentacle, (**I**) 5ht-like immunoreactive perikarya (5ht-p), which are associated with the tentacular nerve ring (tnr) and with the inner nerve ring (inr). Abbreviations: 5ht-afn, two lateroabfrontal neurites exhibited 5ht-like immunoreactivity; 5ht-p (inr), serotonin-like immunoreactive perikarya connected with the inner nerve ring; 5ht-p (tinr), serotonin-like immunoreactive perikarya connected with the tentacular nerve ring; afn, abfrontal neurite bundle; fn, frontal neurite bundle; inr, inner nerve ring; it, inter-tentacular nerves; lfn, laterofrontal neurite bundle; tnr, tentacular nerve ring.

Each tentacle has several anatomical zones: one frontal zone, which faces the mouth; two laterofrontal zones; and one abfrontal zone, which is opposite to frontal zone (Fig. 6A,B). The abfrontal zone is innervated by thin neurites that are grouped into 7–8 neurite bundles (Fig. 7A,C). Neurites of the abfrontal side extend from the perikarya of the tentacular nerve ring (Fig. 7E,H). There are inter-tentacular empty spaces between neurite bundles of adjacent tentacles (Fig. 7A). Among the abfrontal neurite bundles, the two most lateral groups of neurites exhibit serotonin-like immunoreactivity (Fig. 7E,I). According to transmission electron microscopy (TEM), each abfrontal neurite bundle consists of 4–11 neurites of small diameter and with electron-dense cytoplasm (Fig. 6G). Each laterofrontal zone is innervated by a thick neurite bundle, which extends from T-like inter-tentacular nerves. Two inter-tentacular nerves emanate from the inner nerve ring, extend between adjacent tentacles at their base, fuse with each other, and give rise to the two laterofrontal nerves, which penetrate into adjacent tentacles (Fig. 7B–D). Each laterofrontal nerve consists of about 20 thin neurites with electron-dense cytoplasm and one thick neurite with electron-light cytoplasm (Fig. 6H). The frontal zone is innervated by a thick, compact neurite bundle, which extends from the inner nerve ring (Fig. 6B). The frontal neurite bundle extends from the inner nerve ring by two or three thin projections (Fig. 7C,D,G).

Between the bases of adjacent tentacles, there are large, inner 5ht-lir perikarya (Fig. 7E,F,I). These perikarya are flask-shaped and associated with the inner nerve ring (Fig. 3D,E). The size of inner 5ht-lir perikarya is comparable with size of multipolar 5ht-lir perikarya, which are associated with the tentacular nerve ring (Fig. 7I). According to TEM, large cells between tentacles form outer and inner rows (Fig. 6C,E). Cells of the inner row can be regarded as neuronal because their cytoplasm contains synaptic vesicles (Fig. 6F). These cells are surrounded by numerous neurites of the inner nerve ring (Fig. 6F).

In each tentacle, there are several subperitoneal neurites, which extend between the basal matrix and peritoneal cells of the coelomic lining. Subperitoneal neurites are characterized by light cytoplasm and the presence of numerous longitudinal microtubules (Fig. 6D).

## Discussion

### Morphology of the lophophore in phoronids

In adult phoronids, there are four main types and several transitional types of lophophore^3,15^. The type of lophophore usually depends on body size^15,25^. The simplest is the oval lophophore, which is typical for the very small *Phoronis ovalis* and for juveniles of some species. The horseshoe-shaped lophophore is the most common type and occurs in phoronids of intermediate size. The spiral lophophore is characteristic of large phoronids. The largest phoronid *Phoronopsis californica* has a helicoidal lophophore, which is the most complex type.

### Nervous system of the lophophore and tentacles in adult phoronids

**Figure 8:**
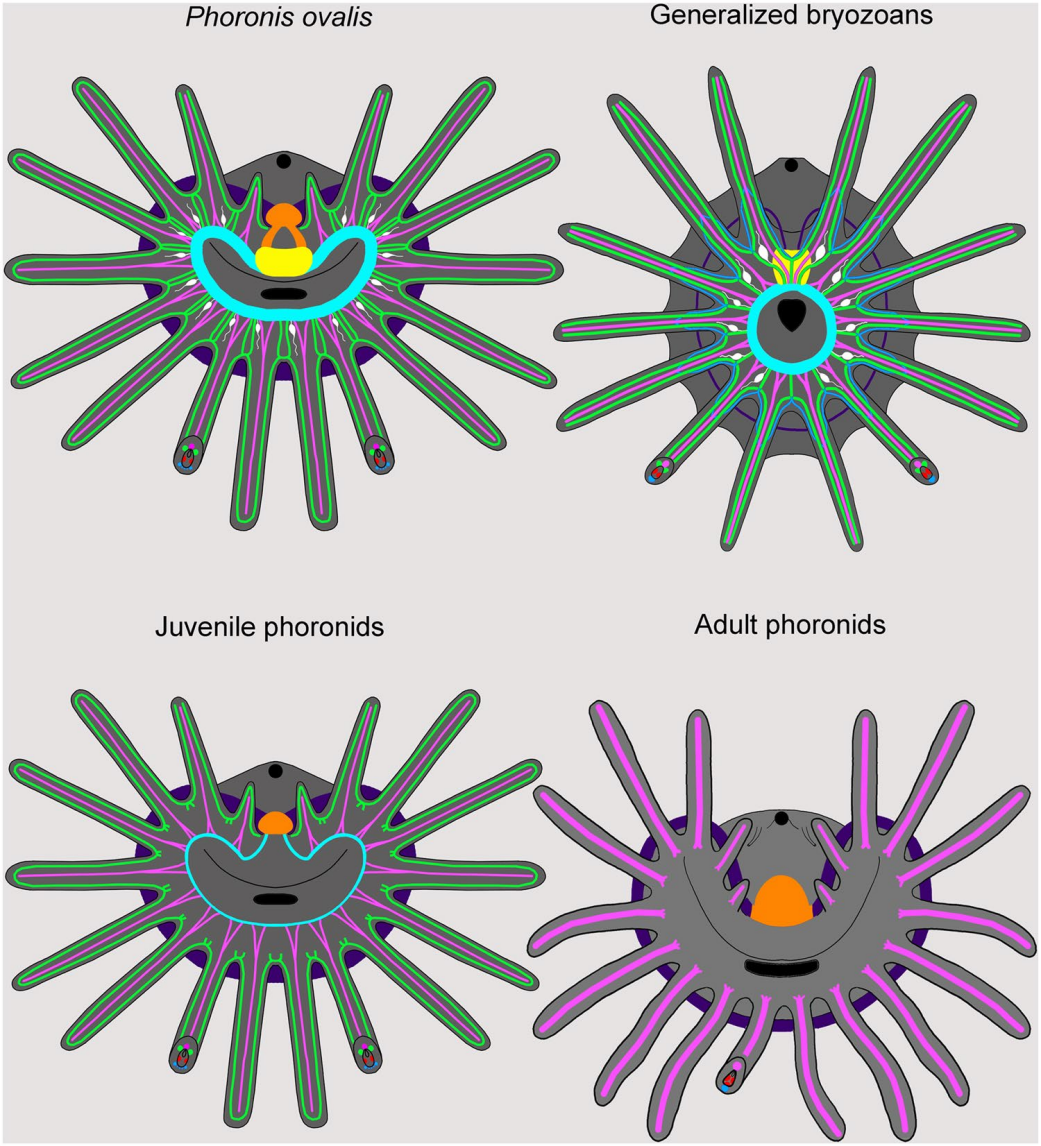
Schemes of the organization of the lophophore nervous system in phoronids and bryozoans. Schemes are based on: *Phoronis ovalis* – herein, juvenile phoronids –^20,21^, adult phoronids –^22–24,26^, generalized bryozoa –^6,17,31–39^. In all schemes, the oral side is to the down, the anal side is at the top. Nerve elements, which are supposed to be homologous, are done in the same color. Color table: yellow – inner nerve center (cerebral ganglion – in bryozoa; and inner ganglion – in *Phoronis ovalis*), orange – dorsal ganglion, cyan – inner nerve ring, magenta – outer nerve ring, green – laterofrontal nerves of tentacles, pink – frontal nerves of tentacle, blue – abfrontal nerves on tentacle, white – inter-tentacular sensory cell, red – subperitoneal neurites, black – mouth and anus.

Nervous system of the lophophore in adult phoronids has seldom been studied by modern methods. The most detailed description of the anatomy of the lophophoral nervous system was made by histological techniques^22,26^. The ultrastructure of the main nerve elements and nervous tracts in tentacles has been studied in several phoronid species^23,24,27,28^. According to all of these reports, the lophophoral nervous system in adult phoronids includes a dorsal nerve plexus (=a dorsal ganglion?), a tentacular nerve ring, and nerve tracts in tentacles (Fig. 8). The dorsal ganglion is located between the mouth and anus, on the dorsal side of the epistome, which is an epidermal fold that covers the mouth. The dorsal ganglion consists mostly of large-diameter perikarya, which apparently are motor neurons^24^. The tentacular nerve ring passes along the outer side of the lophophore at the tentacle bases and repeats the shape of the lophophore. The tentacular nerve ring consists mostly of transversal neurites and small-diameter perikarya, which are apparently sensory^23,24^. Based on histology and TEM, the tentacles are innervated by two nerve tracts, which extend along the frontal and abfrontal sides^24,28,29^ (Fig. 8). These nerves are thought to extend from the tentacular nerve ring^22,26^. The frontal and abfrontal tentacular nerve tracts develop similarly in terms of the number of bundles and number of axons per bundle^28^. At the same time, several large-diameter neurites occur in the frontal nerve tract^24^. Specific laterofrontal sensory cells are associated with the frontal nerve tract^23,27^.

According to recent reports concerning juvenile phoronids^20,21^, innervation of the lophophore in juveniles differs from the traditional scheme described above for the lophophore in adults (Fig. 8). In addition to the dorsal ganglion and the tentacular nerve ring, the oval lophophore of *Phoronopsis harmeri* juveniles (whose adults have the spiral type of lophophore) is innervated by the minor (=inner) nerve ring, which extends along the inner side of the lophophore^20^. Compared to the tentacular nerve ring, the inner nerve ring of *P. harmeri* juveniles is weak, consists of several neurites, and is not associated with prominent perikarya. In *P. harmeri* juveniles, each tentacle is innervated by six nerve tracts: one frontal, two laterofrontal, two lateroabfrontal, and one abfrontal. The frontal nerve tract extends from the inner nerve ring; the laterofrontal nerve tracts seem to be unconnected with any nerves of the lophophore. Two lateroabfrontal nerve tracts extend from the inter-tentacular nerves, which originate from the tentacular nerve ring and extend between the tentacle bases. Inter-tentacular nerves, which the juvenile inherits from the larva, are very thin^20^. The abfrontal nerve tract extends from both the tentacular nerve ring and the inter-tentacular nerves^20^.

In *Phoronis ovalis* adults, the nervous system of the lophophore looks more similar to that of the juveniles than adults of other phoronid species (Table 1). This similarity results from the presence of the inner nerve ring and the innervations of tentacles in *P. ovalis* adults. As opposed to the inner nerve ring оf juvenile *P. harmeri*, the inner nerve ring in *P. ovalis* adults is very strong, consists of many neurites, and is associated with prominent 5ht-lir perikarya. The presence of an inner ganglion seems to be a unique feature of *P. ovalis* adults, i.e., it has not been previously described in any phoronid.

**Table 1:** Nerve elements of the lophophore and tentacles in phoronids and bryozoans (see Fig. 8).

Nerve elements of the lophophore and tentacles	Phoronida adults^22–24,26^	Phoronida juveniles^20,21^	*Phoronis ovalis* adults	Bryozoa^6,17,31–39^
Presence of the nerve centre on the dorsal side оf the body	+	+	+	+
Presence of the nerve centre on the oral side of the epistome	−	−	+	−
Nerve alоng the frontal side of tentacles (inner nerve of the lophophore)	−	+inner nerve ring	+inner nerve ring	+circumoral nerve ring
Intensity (thickness) of the inner nerve of the lophophore	−	thin	thick	thick
Nerve alоng the abfrontal side of tentacles (outer nerve of the lophophore)	+tentacular nerve ring	+tentacular nerve ring	+tentacular nerve ring	+in some ctenostomes (outer nerve) — in most of bryozoans
Innervation оf frontal side of tentacles	?	from inner nerve	from inner nerve	from inner nerve
Innervation оf laterofrontal sides of tentacles	from outer nerve	?	from inner nerve	from inner nerve
Presence оf T-like (inter-tentacular) neurite bundles	?	+	+	+
Presence of inter-tentacular 5ht-lir perikarya	?	−	+	+

“+” Indicates presence; “−” indicates absence; “?” indicates that information is lacking.

The current report provides the first detailed description of the innervation of tentacles in adult phoronids. The new morphological observations of *P. ovalis* adults are similar to our previous observations of juvenile phoronids (Table 1). The tentacles of some adult phoronids contain subperitoneal neurites (axons?)^28,30^. These neurites run between the peritoneal cells and the basement membrane. The subperitoneal neurites are large in diameter and have electron-lucent cytoplasm and thick, longitudinal microtubules. Fibres with a similar ultrastructure were found in the tentacles of *P. ovalis* adults in the current study.

Overall, the nervous system of the lophophore seems to have more elements or components in *P. ovalis* adults than in the adults of other phoronids. A complex (multicomponent) organization of the nervous system of the lоphophore was described in the articulate brachiopod *Hemithiris psittacea* ^19^ and was suggested to be the ancestral organization for all lophophorates.

### Nervous system of the lophophore and tentacles in bryozoans

The lophophore nervous system and the innervation of tentacles in bryozoans from all taxa except the Stenolaemata has been described in many studies that used light microscopy, immunoсytochemistry combined with laser confocal microscopy, and TEM (e.g. refs^17,31–37^).

In the organization of the bryozoan nervous system, there are variations, which concern the structure of the cerebral ganglion, the innervation of tentacles, and the connections between tentaсular neurite bundles and the main nerve centers. The results were reviewed in recent papers that presented opinions about general patterns of the localization of the main nerve tracts in lophophores in all bryozoans^34,36,37^. All of these reports indicate that the bryozoan lophophore has a cerebral ganglion and an inner nerve ring (=circumoral nerve ring) (Fig. 8). According to recent results, ctenostome bryozoans have an outer nerve ring extending along the outer side of the lophophore^6^. In all bryozoans studied to date, there are large 5ht-lir perikarya associated with the circumoral nerve ring and located between tentacles at their bases. The number of perikarya is strictly established, and for each species is equivalent to the number of tentacles minus 2. These perikarya connect the circumoral nerve ring or the cerebral ganglion via prominent thick nerves. Large 5ht-lir perikarya appear to be sensory: they contact the surface of the epithelium and bear “serotonincilia”^6,17^. According to comparative analysis of data obtained from many species, the 5ht-lir perikarya do not form a circle and are arranged into oral and anal groups^17^. In most phylactolaemates, which usually have a large number of tentacles, there are many bipolar 5ht-lir perikarya scattered between the tentacles at their bases^36^.

Bryozoans have two to six longitudinal neurite bundles in each tentacle. Most phylactolaemates have six basiepidermal tentacular nerves: mediofrontal, medioabfrontal, one pair of lateroabfrontal, and one pair of laterofrontal^36^. Gymnolaemates usually have four tentacular nerves: one abfrontal, one mediofrontal, and one pair of laterofrontal^35,38^. Some ctenostome bryozoans have two tentacular nerves: the frontal, which originates as a result of the fusing of two laterofrontal and one frontal neurite bundles, and the abfrontal^6^. Accordingly to their origin, two main types of tentacular nerves can be established in bryozoans. The first type, which occurs in phylactolaemates, is characterized by the origin of all tentacular nerves from inter-tentacular (radial) nerves^36^. In the second type, which is common in gymnolaemates, some nerves originate from inter-tentacular nerves, and others originate directly from the circumoral nerve ring^32,35,38^. Many bryozoans have basiperitoneal tentacular nerves^6,35,39^, which do not show immunoreactivity against acetylated alpha tubulin and thereby cannot be regarded as nerve elements^35^.

### Оrganization of the nervous system of the lophophore and tentacles in *Phoronis ovalis* and bryozoans

Because detailed data on early development are lacking, it is impossible to strictly establish the homology between some parts of the lophophore, including the nerve elements, in *P. ovalis* and bryozoans. For this reason, this report only suggests the possible homology between nerve elements of the lophophore in *P. ovalis* and bryozoans based on their location in respect to the same organs (mouth, tentacles, and epistome, if present) in both taxa.

As indicated in Table 1, many features of the organization of the lophophore nervous system seem similar in bryozoans and *P. ovalis*. The most prominent similarity is the presence of a conspicuous inner nerve ring (circumoral nerve) extending around the mouth and along the frontal side of the tentacles. This nerve ring is strongly developed in both bryozoans and *P. ovalis*. A second similarity is that the circumoral nerve ring in both bryozoans and *P. ovalis* is associated with 5ht-lir perikarya, which are located between tentacles at their bases (Fig. 8). The connection between the laterofrontal tentacular nerves and the innerve nerve ring is the third similarity between the lophophore nervous system in bryozoans and *P. ovalis*. A fourth similarity is that both bryozoans and *P. ovalis* have an outer lophophoral nerve extending along the abfrontal side of the tentacles; this nerve is represented by the tentacular nerve ring in *P. ovalis* and by the outer nerve in some ctenostome bryozoans.

### Hypothetical scenario of evolution of bryozoans from a phoronid-like ancestor

That bryozoans and phoronids may be closely related has been indicated by many authors, who have suggested that bryozoans and phoronids are sister groups that originated from protophoronids or that bryozoans developed from phoronids^1,13,40–50^. The current data also suggest that both phoronids and bryozoans originated from protophoronids. This inference is inconsistent, however, with recent molecular phylogenetic data, which indicate that bryozoans are a separate clade on the phylogenetic tree of Bilateria and that phoronids form a clade (the Brachiozoa) with brachiopods^9,51^. According to the molecular data, phoronids and bryozoans are not relatives, and the lophophore has been evolved twice among bilaterians: in the Brachiozoa and in the Bryozoa. On the other hand, similarities in lophophore organization and in the general body plan are consistent with the inferences that phoronids and bryozoans are closely related and that the lophophorates is monophyletic^5,6,18^. As noted earlier, the molecular data are inconsistent with the morphological data. At the same time, the position of the Bryozoa on the phylogenetic tree of the Bilateria is still ambiguous and apparently depends on which molecular data are used in the analysis (for a review, see ref.^52^). Clarification of bryozoan phylogeny will require both new morphological data and new molecular data.

**Figure 9:**
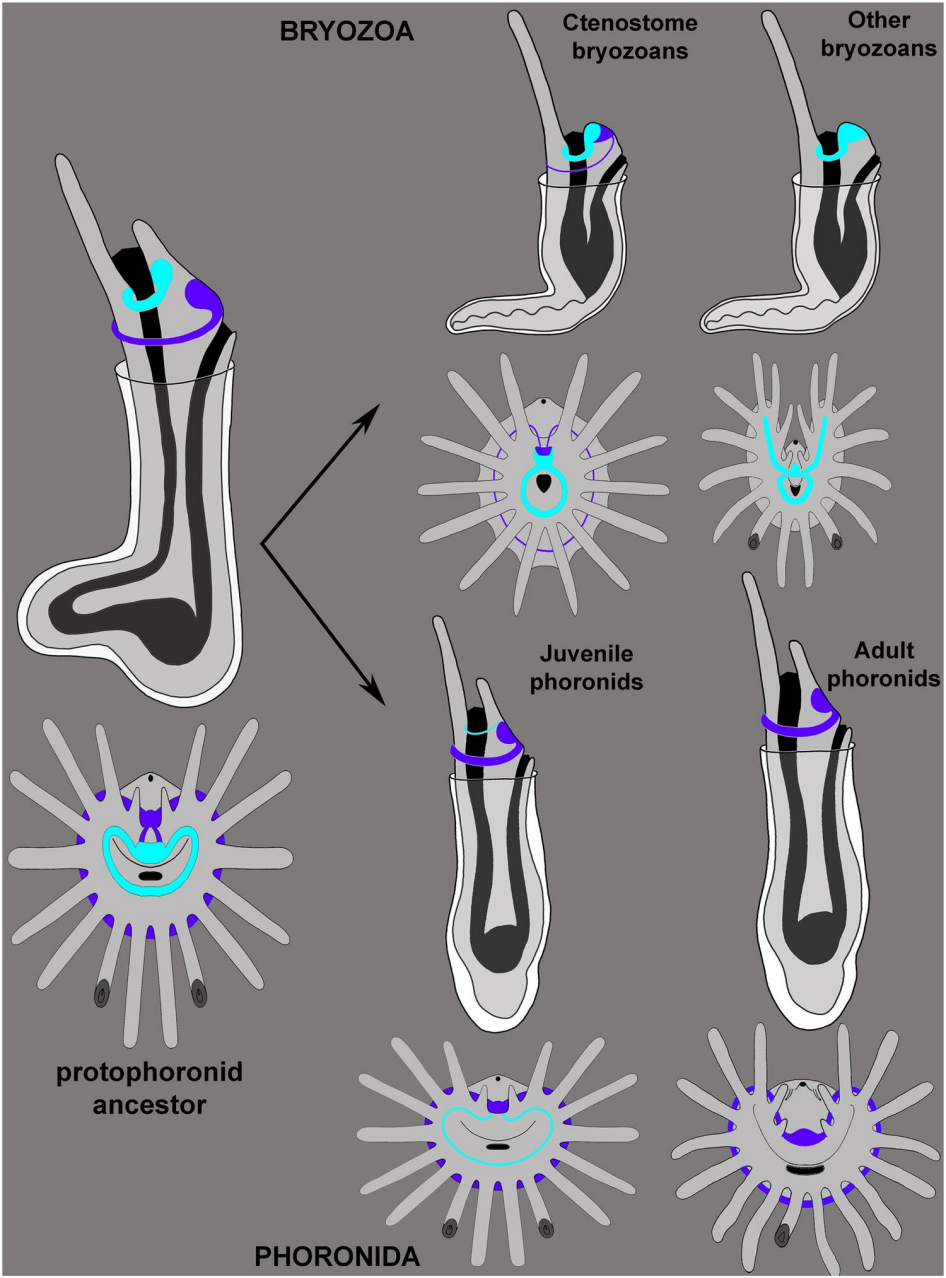
Hypothetical scenario of evolution of the lophophore nervous system in phoronids and bryozoans. Protophoronid ancestor had two nerve centres and two nerve rings: inner (cyan) and outer (purple). In bryozoans, these nerve centres fused each other forming cerebral ganglion, which exhibits zonality. The outer nerve ring underwent reduction and had lost. In phoronids, inner nerve centres and inner nerve ring were reduced in adults, but retained in *Phoronis ovalis* and maintained in part in juvenile phoronids.

Because phoronids and bryozoans have a similar body plan, which seems to be unique for these two groups, it is logical to suspect that this plan was inherited by both groups from a common ancestor. Several previous papers have suggested that the body plan of phoronids probably evolved to enable the organism to escape from danger by digging into a soft substratum^20,53^. This change in body plan involved the formation of a large ventral protrusion, the shortening of the dorsal side, and the appearance of a U-shaped gut. This ancestor may have given rise to two evolutionary stems: the phoronids and bryozoans. According to this hypothetical scenario, the ancestor shared many features with recent *P. ovalis* adults: it was small and wrapped in a chitinous envelope, had asexual reproduction, and had a simple lophophore that was innervated by two ring nerves: the inner and the outer (Fig. 9).

In the phoronid stem (Fig. 9), the lophophore increased in complexity, the body and epistome became larger, the inner nerve center and the inner nerve ring were reduced, asexual reproduction was mostly lost, and a tube formed at the base of the chitinous envelope. According to this possibility, *P. ovalis* seems to be the most primitive phoronid, whose organization maintains many ancestral features. The basal position of *P. ovalis* among other phoronids is suggested by both morphology^4^ and molecular phylogeny^16^. On the other hand, *P. ovalis* may retain primitive features because of its neotenic origin, which is suggested by cladistic analysis of phoronid taxonomy^54^.

In the bryozoan stem (Fig. 9), the protophoronid ancestor underwent simplification of the lophophore, an extreme reduction in body size and a complete reduction of the epistome (which led to the fusion of the inner and outer nerve centers), a great reduction in the outer nerve ring, a great increase in asexual reproduction, and the formation of a cystid at the base of the chitinous envelope. According to this possibility, recent bryozoa species with a “soft” chitinous cystid and with a simple lophophore with 16–20 tentacles but without an epistome should be regarded as the most primitive. Ctenostome bryozoans best fit this description. In addition, ctenostome bryozoans retained the outer nerve ring, and their cerebral ganglion has zonality, which may have resulted from the fusion of several nerve centers^6^. According to some data, ctenostome bryozoans appeared first in the paleontological history of bryozoans. These were burrowing, non-skeletal colonial animals, whose traces are known from marine sediments beginning with the Early Ordovician^55,56^. Paleontological and morphological data support the basal position of the ctenostome among bryozoans. On the other hand, most phylogenetic data indicate the basal position of fresh water phylactolaemates among bryozoans^10,12,57^. At the same time, phylactolaemates have several apomorphic features: for example, their horseshoe-spade lophophore developed *de novo* due to appearance of inner rows of tentacles^17^. Like the new lophophore, the epistome of phylactolaemates may have developed *de novo* but may also have been inherited from a phoronid-like ancestor.

## Materials and Methods

### Sampling of animals and light microscopy

Specimens of *Phoronis ovalis* Wright, 1956 are embedded into shells of bivalve *Pecten* sp., which were collected in Onezhskiy Bay, White Sea in July 2016 by SCUBA diving at 3–10 m depth. Animals were extracted from the shells and photographed in the laboratory using a Leica M165C (Leica, Germany) stereomicroscope equipped with a Leica DFC420 digital camera.

### Immunocytochemistry

Whole animals were fixed in a 4% paraformaldehyde solution in filtered sea water and washed in phosphate buffer (pH 7.4) (Fisher Scientific, Pittsburgh, PA, USA) with Triton X-100 (1%) (Fisher Scientific) (PBT) for a total of 24 h. Nonspecific binding sites were blocked with 12% normal donkey serum (Jackson ImmunoResearch, Newmarket, Suffolk, UK) in PBT overnight at 4 °C. The specimens were then transferred into primary antibody, which was a mixture of anti-α-Tubulin-mouse (1:600) and anti-serotonin-rabbit (1:1100) (ImmunoStar, Hudson, WI, USA) in PBT, and incubated for 24 h at 4 °C with rotation. Specimens were washed in PBT and were then exposed to the secondary antibody, which was 532-Alexa-Rabbit (1:1000) and 635-Alexa-Mouse (1:1000) (Invitrogen, Grand Island, NY, USA) in PBT, for 24 h at 4 °C. After washing in phosphate buffer specimens were embedded in Murray Clear. Specimens were viewed with a Nikon Eclipse Ti confocal microscope (Moscow State University, Moscow, Russia). Z-projections were done using Image J version 1.43 software. Three-dimensional reconstructions were produced in Amira version 5.2.2 software (Thermo Fisher Scientific, MA, USA). Z-projections and TEM micrographs were processed in Adobe Photoshop CS3(Adobe World Headquarters, San Jose, California, USA).

### Transmission electron microscopy (TEM)

For TEM, the animals were fixed in 2.5% glutaraldehyde in phosphate buffer saline and were postfixed for 2 h in 1% osmium tetroxide in the same buffer saline. After the specimens were washed with the same buffer saline, they were transferred through an ethanol series and stored in 70% ethanol at 4 °C. Further preparation included dehydration in an ethanol series and acetone, and embedding in Spurr resin (Sigma). Semithin and thin sections were cut with a Leica UC-6 ultratome (Leica, Germany), then were stained with methylene blue, observed with a Zeiss Axioplan2 microscope, and photographed with an AxioCam HRm camera. Ultrathin sections were stained in uranyl acetate followed by lead nitrate and were examined with JEM-1011 JEOL and JEM-100 B-1 JEOL transmission electron microscopes (JEOL, Akishima, Japan).

### Scanning electron microscopy (SEM)

Scanning electron microscopy (SEM) For SEM, the animals were fixed in 2.5% glutaraldehyde in phosphate buffer (PBS) Fixed animals had been washed in PBS and then dehydrated in ethanol followed by an acetone series were critical point dried and then sputter coated with platinum-palladium alloy.Specimens were examined with a CamScan S2 scanning electron microscope.

### Data availability statement

The data sets analyzed during the this study are available from the author in response to requests

### Ethics statement

The field sampling did not involve endangered or protected species. The use of phoronids in the laboratory does not raise any ethical issues.
